# The association of social media use and other social factors with symptoms of attention-deficit/hyperactivity disorder in Egyptian university students

**DOI:** 10.1186/s12888-024-05988-6

**Published:** 2025-01-07

**Authors:** Mustafa Mohammed Hassan, Hisham Ahmed Orebi, Basem Salama, Ibrahim Ali Kabbash

**Affiliations:** 1 Faculty of Medicine, Tanta University, Tanta, Egypt; 2 Professor of Family & Community Medicine, Faculty of Medicine, Northern Border University, Arar, Saudi Arabia; 3 Public Health & Community Medicine, Faculty of Medicine, Tanta University, Tanta, Egypt

**Keywords:** Social media, University, Students, ADHD, Prevalence, Risk

## Abstract

**Background:**

Attention-Deficit/Hyperactivity Disorder (ADHD) is one of the most common neurodevelopmental disorders of childhood. Many studies reported that excessive social media use is more likely to develop symptoms of ADHD.

**Methods:**

A cross-sectional study was carried out at Tanta University. The study recruited a total sample of 933 college students from Tanta University from five randomly selected colleges. Data was collected using self-administrated questionnaires made in Google Forms sent to social media groups of students. We used the Adult ADHD Self-Report Scale (ASRS-v1.1) Symptom Checklist instructions.

**Results:**

The total number of respondents was 933. Those at risk of ADHD represented 30.5%. All sociodemographic variables were not found to significantly affect the risk for ADHD except for the presence of the father in the family. This of ADHD significantly increased to 42.3% and 44.4% for those whose fathers were traveling abroad or separated from their mothers, respectively (*p* = 0.031). The risk for ADHD significantly decreased among those who practice sports to reach 24.1% compared to 33.8% for those who did not (*p* = 0.002). The risk for ADHD increased significantly among those who used mass media to watch reels. ADHD risk was found to increase with increased hours of watching media during the study period (*p* < 0.001) during weekends (*p* = 0.001) and holidays (*p* = 0.038). Multivariate analysis showed that practicing sports independently reduces the risk for ADHD (Exp B = 0.679). Meanwhile, both watching reels and hours of using media during the study period independently increase the risk (Exp B = 1.493 and 1.390, respectively).

**Conclusion:**

There are many factors affecting ADHD, we found that watching reels, status, stories, and shorts and the number of hours spent on social media during the study period are independent risk factors. Practicing sports is an independent protective factor. Most of the factors need further studies.

## Background

Attention deficient/hyperactivity disorder (ADHD) is one of the most common neurodevelopmental disorders of childhood. It may last into adolescence or adulthood. Patients with ADHD may have trouble paying attention, controlling impulsive behaviors, or being overly active [1]. Therefore, it can be a challenge in academic or occupational life. ADHD is not caused by a single cause or risk factor which exposure to it means getting ADHD. ADHD is a multifactorial disorder, which has genetic and environmental factors [2]. ADHD runs in families and a study showed that later-born siblings of those with ADHD or autism spectrum disorder (ASD) had an increased risk of ADHD and ASD [3].

There are many environmental factors during prenatal, perinatal, and postnatal periods like maternal smoking and alcohol consumption during pregnancy, prenatal exposure to illegal drugs and antidepressant drugs, maternal psychological conditions before and during pregnancy, such as anxiety, depression, and sleep disorders, low birth weight, prematurity, post maturity, complications during pregnancy and delivery, postnatal jaundice, childhood stroke, lead exposure and traumatic brain injury [4–7]. Some studies stated that inflammations locally or peripherally are associated with some psychiatric diseases, including ADHD. Studies stated that factors like oxidative stress, neuro-inflammation, autoimmune diseases, maternal immune activation, allergic diseases, and other immune-mediated conditions are linked to ADHD, but there are limitations in some of these studies such as a small sample size, a lack of follow-up in different stages of the disease and a lack of correlation with disease severity [2].

A systematic review and meta-analysis study that included 175 studies about the prevalence of ADHD, found that the overall pooled estimate is 7.2% (95% confidence interval: 6.7–7.8%) [8]. A study conducted at Tanta University, Egypt in 2011 found the prevalence of ADHD was 2.66%. So, ADHD is not uncommon among college students [9]. In this age, which is characterized by digital interconnectedness and information dissemination, social media use is one of the most popular online activities. There are 4.76 billion social media users around the world, which is just less than 60% of the total global population in January 2023 [10]. There were 46.25 million social media users in Egypt in January 2023 which equals 41.4% of the total population of Egypt [11].

Many studies reported that the more social media use the more possible to develop symptoms of ADHD [12–14]. The correlation between problematic social media use and ADHD was demonstrated (12). In a study where adolescents were followed up over two years, there was a statistically significant but modest association between higher frequency of digital media use and subsequent symptoms of ADHD [15]. Another study found that over time, problematic social media use increased ADHD symptoms. However, social media use intensity did not increase ADHD. That study found that problematic social media use increased attention deficit and impulsivity which may be due to the attractiveness of social media platforms which makes it harder to resist using these platforms. This relation was unidirectional as the reverse pattern was not detected [16]. Therefore, there is evidence that problematic social media use has harmful effects on users [17, 18]. The impact of social media use on ADHD symptoms was not driven by the frequency of use, but rather by the addiction-like aspect of problematic use, such as the constant urge to go online or the inability to control social media use, social media use intensity may be a normative behavior in adolescents’ daily lives [16].

Studies recognized that social media use is related to ADHD symptoms, but no research studied the relationship between the pattern of use of social media and ADHD, which is important to find out to increase college students and adolescents’ awareness to control the prevalence of ADHD and to preserve the health of adolescents and college students. This study was conducted at Tanta University to investigate the association between social media use and symptoms of Attention-Deficit/Hyperactivity Disorder (ADHD) among university students in Egypt. We also explored the potential influence of other social factors on ADHD symptoms.

## Methods

This cross-sectional study was carried out for six months from January 2023 to June 2023 at Tanta University. Tanta University is an Egyptian university located in Tanta City, Gharbia Governorate in the Middle of the Nile Delta. It includes 16 faculties and one technical institute. The university in the academic year 2022\2023 hosted 121,279 students.

### Study objectives

- Investigate the association between social media use and ADHD symptoms among university students.
- Assess the potential relationship between other social factors and ADHD symptoms.

The sample size and power analysis were calculated using the Epi-Info software statistical package created by the World Health Organization and Center for Disease Control and Prevention, Atlanta, Georgia, USA version 2002. The criteria used for sample size calculation were as follows:

- 95% confidence limit.
- The estimated number of students showing ADHD manifestations is at 35% with a margin of error of 5%. A design effect of 2.5 was added to compensate for non-randomization.
- The sample size based on the previously mentioned criteria was found at *N* = 873.
- We increased the sample size to 933 to compensate for missed information and improve the quality of the data of the study.

The faculties of Tanta University were stratified into three groups: medical faculties, practical faculties, and humanitarian studies faculties. The study population included students from randomly selected faculties representing each stratum namely, Medicine, Pharmacy, Engineering, Science, and Arts which include 7131, 3028, 6930, 4339, and 25,285 students in the academic year 2022\2023, respectively. The first two faculties are medical ones. The second two faculties are practical ones but the fifth one is humanitarian studies faculty.

The total sample selected was 933 college students from Tanta University distributed as 175 students from the Faculty of Medicine, 197 students from the Faculty of Pharmacy, 182 students from the Faculty of Science, 180 students from the Faculty of Engineering, and 199 students from the Faculty of Arts. The faculties were selected randomly by stratified sampling techniques to represent studies from different academic backgrounds. A nonrandomized convenient sample was next selected from each faculty.

Data were collected via self-administrated questionnaires made in Google Forms and sent to social media groups of students. The target was almost 200 students from each selected faculty during the study period. The questionnaire includes 64 questions distributed as 17 sociodemographic questions, 7 questions for social media use, 18 questions for Adult ADHD Self-Report Scale (ASRS-v1.1) Symptom Checklist instructions, 21 questions for Modified Social Media Disorder Scale and a question for previous ADHD diagnosis. The Adult ADHD Self-Report Scale is primarily used to identify adult ADHD in primary care settings. The Symptom Checklist is an instrument consisting of the eighteen DSM-IV-TR criteria. Six of the eighteen questions were found to be the most predictive of symptoms consistent with ADHD. These six questions are the basis for the ASRS v1.1 Screener and are also Part A of the Symptom Checklist. Part B of the Symptom Checklist contains the remaining twelve questions and gives more insight into symptoms. No total score or diagnostic likelihood is utilized for the twelve questions. The six questions in Part A are the most predictive of the disorder and are best for use as a screening instrument [19].

The collected data were organized, tabulated, and statistically analyzed using SPSS version 19 (Statistical Package for Social Studies) created by IBM, Illinois, Chicago, USA. For numerical values the range, mean, and standard deviations were calculated. The differences between the two mean values were used using the student’s t-test. For categorical variables, the number and percentage were calculated and differences between subcategories were tested by Chi-square test. The multivariate analysis was done sung logistic regression. The level of significance was adopted at *p* < 0.05.

### Ethical considerations

The Ethical Committee of Scientific Research at Tanta Faculty of Medicine approved our application before the beginning of the study (approval code: 36121\11\22). An informed consent of the participants in the study was obtained by adding a statement at the beginning of the questionnaire about the purpose of the study and asking participants to complete the sheet if consenting to share. Data was not used for other purposes than scientific research. Confidentiality and privacy were guaranteed during the whole period of the study.

## Results

Our study included 933 students; 285 students were at risk of ADHD (30.55%) which was calculated according to part A of the Adult ADHD Self-Report Scale. Four students of those who had a risk of ADHD had only no risky questions in part B of the Adult ADHD Self-Report Scale (1.4%). Meanwhile, 46 students who had the risk had 1–3 risky questions in part B of the Adult ADHD Self-Report Scale (16.1%). It was clear that those at risk of ADHD tended to have a greater number of risk questions in part B of the Adult ADHD Self-Report Scale. Among those at risk of ADHD, 94 students had from 7 to 9 risky questions in part B of the Adult ADHD Self-Report Scale (33%). Among those with a risk of ADHD, those with > 6 risky questions represented 44.5% compared to only 9.5% among those who were not at risk for ADHD. The distribution of studied students by risky questions in part B of the adult ADHD self-reported scale showed significant differences. (*p* < 0.001) (Table 1).

**Table 1:** Distribution of studied students by risky questions in part B of the adult ADHD self-reported scale

Number of risky questions in List B	Not at risk (648)		At risk of ADHD (*n* = 285)	
	*n*	%	*n*	%
**None**	56	8.6	4	1.4
1–3	295	45.6	46	16.1
4–6	235	36.3	114	40.0
7–9	54	8.3	94	33.0
10–12	8	1.2	27	9.5
*X* ^*2*^	175.767			
p	< 0.001*			

*Significant

There was no statistically significant difference between the two groups, those who had the risk and those who didn’t have it, concerning the mean age (20.11 *±* 1.51 and 20.28 *±* 1.58, respectively). There is no statistically significant association between risk for ADHD and gender, parents’ education, parents’ jobs number of siblings, and birth order. Meanwhile, the absence of a father from the family was associated with an increased risk of ADHD. Students whose fathers were traveling abroad or separated from their mothers represented the highest percentage among those with ADHD (42.3% and 44.4%, respectively) (*p* = 0.031) (Table 2).

**Table 2:** Characteristics of studied students

Variables	Not at risk (648)		At risk of ADHD (*n* = 285)		t/X^2^	*p*
	*n*	%	*n*	%		
**Age in years**						
Mean ± SD	20.28 ± 1.59		20.11 ± 1.51		1.526	0.127
**Sex**:						
Males	244	71.1	99	28.9	0.725	0.395
Females	404	68.5	186	31.5		
**Fathers’ education**:					1.733	0.630
Illiterate	18	75.0	6	25.0		
Primary	51	75.0	17	25.0		
Secondary	128	67.4	62	32.6		
University	451	69.3	200	30.7		
**Mothers’ education**:					4.736	0.192
Illiterate	38	82.6	8	17.4		
Primary	41	69.5	18	30.5		
Secondary	161	66.5	81	33.5		
University	408	69.6	178	30.4		
**Fathers’ job**:					3.177	0.529
Unemployed	25	67.6	12	32.4		
Manual worker	62	68.9	28	31.1		
Employee	132	67.3	64	32.7		
Professional	260	68.1	122	31.9		
Private	169	74.1	59	25.9		
**Mothers’ job**					8.162	0.086
Housewife	352	70.7	146	29.3		
Manual worker	10	47.6	11	52.4		
Employee	61	70.9	25	29.1		
Professional	193	67.0	95	33.0		
Private work	32	80.0	8	20.0		
**Presence of father in the family**:					10.670	0.031*
Present	516	70.4	217	29.6		
Traveling abroad	45	57.7	33	42.3		
Died	77	74.8	26	25.2		
Separated from mother	10	55.6	8	44.4		
Prisoned	0	0.0	1	100.0		
**Number of siblings**:					1.313	0.189
Range	0–9		0–9			
Mean ± SD	2.55 ± 1.77		2.65 ± 1.13			
**Birth order**					0.023	0.988
Eldest	275	69.6	120	30.4		
Intermediate	235	69.3	104	30.7		
Youngest	138	69.3	61	30.7		

*Significant

Regarding practicing sports and hobbies, this study found a statistically significant difference in the risk of ADHD among those practicing sports (24.1%) as compared to those who did not (33.8%) (*p* = 0.002). There is no statistically significant difference in the frequency of sports practicing/week, having a hobby, and frequency of hobby practicing/ week (Table 3).

**Table 3:** Distribution of studied students by practicing sports and hobbies

Variables	Not at risk (648)		At risk of ADHD (*n* = 285)		X^2^	*p*
	*n*	%	*n*	%		
**Practicing sports**					9.283	0.002*
None	409	66.2	209	33.8		
Yes	239	76.1	76	24.1		
**Frequency of sport practicing/week**:					1.225	0.747
1–2	30	71.4	12	28.6		
3–4	46	79.3	12	20.7		
5–6	102	77.3	30	22.7		
Daily	61	73.5	22	26.5		
**Having a hobby**					0.774	0.379
None	280	68.0	132	32.0		
Yes	368	70.6	153	29.4		
**Frequency of hobby practicing/ week**:					1.871	0.600
1–2	103	74.1	36	25.9		
3–4	37	66.1	19	33.9		
5–6	83	72.2	32	27.8		
Daily	145	68.7	66	31.3		

There is no statistically significant difference in the time when participants started using social media. For the time mostly used on social media, there is no statistically significant difference among those who chose to scroll down and watch long videos. There is a statistically significant difference among those who chose watching reels, status, and stories, chatting with friends, and searching for information. Those who chose watching reels (38.4%) (*p* = 0.001), those who chose chatting with friends (24.4%) (*p* = 0.050), and those who chose searching for information (19%) (*p* = 0.008). There is a statistically significant difference in hours using social media during study periods, weekends, and holidays. For those who spent less than 2 h in the study, the prevalence of risk of ADHD was 21% and increased to 37.6% among those who spent > 4 h (*p* < 0.001). The same observation was found among those who spent more hours on social media during weekends (34.8%) (*p* = 0.001) and those who spent > 4 h during holidays (32.7%) (*p* = 0.038) (Table 4).

**Table 4:** Distribution of studied students by their use of social media

Variables	Not at risk (648)		At risk of ADHD (*n* = 285)		X^2^	*p*
	*n*	%	*n*	%		
**Since when starting using media in years**:					2.502	0.475
< 1	13	72.2	5	27.8		
1-<3	109	70.8	45	29.2		
3–5	132	65.0	71	35.0		
> 5	394	70.6	164	29.4		
Time mostly used on media: *						
Scrolling down	196	67.4	95	32.6	0.944	0.331
Watching reels (279)	172	61.6	107	38.4	11.668	0.001*
Watching long videos (198)	142	71.7	56	28.3	0.569	0.451
Chatting with friends (180)	136	75.6	44	24.4	3.826	0.050*
Search for information (*n* = 100)	81	81.0	19	19.0	6.959	0.008*
**Hours using social media during the study period**					18.464	< 0.001*
< 2	128	79.0	34	21.0		
2–4	266	73.1	98	26.9		
> 4	254	62.4	153	37.6		
**Hours using social media during weekends**					13.066	0.001*
< 2	59	76.6	18	23.4		
2–4	208	76.5	64	23.5		
> 4	381	65.2	203	34.8		
**Hours using social media during holidays**					6.546	0.038*
< 2	48	73.8	17	26.2		
2–4	127	77.0	38	23.0		
> 4	473	67.3	230	32.7		

*More than one answer

Multivariate analysis showed that the three significant independent factors affecting ADHD were watching reels (*p* = 0.010) (Exp. B = 1.493), hours using social media during the study period (*p* = 0.011) (Exp. B = 1.390), and practicing sports (*p* = 0.016) (Exp. B = 0.679) which protects from the risk of ADHD (Table 5).

**Table 5:** Regression analysis of variables affecting the risk of ADHD among studied students

Variables	Wald	*p*	Exp. B
The presence of a father in the family	0.515	0.473	1.070
Practicing sports	5.782	0.016*	0.679
Watching reels	6.595	0.010*	1.493
Chatting with friends	1.318	0.251	0.799
Search for information	2.710	0.100	0.637
Hours using social media during the study period	6.435	0.011*	1.390
Hours using social media during weekends	0.401	0.526	1.112
Hours using social media during holidays	0.016	0.898	0.980

## Discussion

Social media has become an integral part of our daily lives, with millions of people using it to connect with others, share information, and stay up to date with the latest news. However, recent studies have raised concerns about the potential negative effects of social media use on mental health, particularly ADHD symptoms among adults. According to a study conducted by Bournemouth University, there is a link between ADHD behaviors and technology addictions among adults, including excessive use of social media and smartphone dependence [20]. Another cross-sectional study conducted among 320 university students in San Diego, CA, USA found that greater social media search for ADHD is related to higher symptom reports among individuals concerned about having ADHD regardless of psychological history [21]. These findings suggest that social media use may exacerbate ADHD symptoms among adults, even in those who have not been diagnosed with the condition.

This study provides valuable insights into the relationship between social media use, ADHD risk, and symptoms among Egyptian university students. Our study found that approximately one-third of the Egyptian Tanta University students included in the study were at risk of ADHD based on the Adult ADHD Self-Report Scale. This prevalence rate aligns with some previous research conducted in different countries. For example, a cross-sectional study conducted among Lebanese adults to measure the association between problematic social media use and ADHD reported that higher problematic social media use (adjusted odds ratio = 1.065) was significantly associated with higher odds of having ADHD [12]. Also, a longitudinal cohort study conducted among 15–16-years-old students in Los Angeles County, California to determine whether the frequency of using digital media is associated with subsequent occurrence of ADHD symptoms during a 24-month follow-up revealed that there was a statistically significant association between higher frequency of digital media use and subsequent symptoms of ADHD [15]. Additionally, the results of a meta-analysis study conducted to assess the relationship between problematic internet use ADHD, and impulsivity were significant [22]. These findings suggest that the prevalence of ADHD risk among university students may be relatively consistent across different cultural and geographical contexts.

Regarding the presence of risky questions in part B of the Adult ADHD Self-Report Scale, our study revealed that a small percentage of students at risk of ADHD did not have any risky questions. This finding is consistent with some prior research that has shown that not all individuals with ADHD exhibit symptoms across all domains or behaviors assessed by the screening measures. In a study conducted in Norway, researchers found that some individuals with ADHD did not endorse symptoms in specific areas, such as impulsivity or inattentiveness [23]. This suggests that the heterogeneity of ADHD symptoms and their presentation is an important factor to consider when assessing and diagnosing the disorder.

In our study, we also observed that a considerable proportion of students who did not have any risk of ADHD showed risky questions in part B of the Adult ADHD Self-Report Scale. This finding is consistent with previous research indicating that some individuals without ADHD may still have some ADHD-related symptoms or behaviors, albeit at a sub-threshold level. A study conducted in the United States among young adults found that individuals without ADHD exhibited a range of ADHD symptoms [24]. In a study conducted in Sweden, researchers found that subthreshold ADHD symptoms were present in approximately 15% of the general population [25]. These findings suggest that ADHD symptoms exist on a continuum, with individuals falling along a spectrum ranging from no symptoms to subthreshold symptoms to clinical ADHD diagnosis. These findings highlight the importance of considering the dimensional nature of ADHD symptoms and the potential overlap between clinical and subclinical presentations [26].

The present study found no statistically significant difference in the mean age between the two groups, those who had the risk of ADHD and those who didn’t. Additionally, there was no significant association between the risk for ADHD and gender, parents’ education, parents’ jobs, number of siblings, and birth order. These findings suggest that age and demographic factors may not play a significant role in the risk of ADHD among Egyptian university students. This finding aligns with a similar cross-sectional study among 2280 undergraduate students from 11 colleges at King Abdulaziz University, Saudi Arabia which also found no significant association between ADHD risk and age or demographic factors [27]. However, a study conducted among 300 young adults in India [28] reported that males and females are significantly different in social media usage which can lead to a conclusion of higher risk to develop ADHD symptoms among females than males.

The present study found that the absence of the father in the family was associated with an increased risk of ADHD. Students whose fathers were traveling abroad or separated from their mothers had the highest percentage of ADHD risk. This finding suggests that family dynamics, specifically the absence of the father, maybe a contributing factor to the risk of ADHD among Egyptian university students. This could be due to a lack of parental authority on how to use social media and for how long, which in turn can lead to social media misuse and the development of ADHD symptoms. There is limited research directly comparing the association between a father’s presence and ADHD risk in university students. However, a study among undergraduate students at King Abdulaziz University in Saudi Arabia [27], reported an increased association between parental divorce and the likelihood of developing adult ADHD.

This study found a statistically significant difference in the risk of ADHD between students who practiced sports and those who did not. Those who practiced sports had a lower risk of ADHD compared to non-practitioners. This finding suggests that engagement in sports may have a protective effect against ADHD symptoms among Egyptian university students. Similar findings have been reported in previous studies. A study conducted on a sample of children and adolescents in Georgia found that participation in organized sports was associated with reduced ADHD symptoms [29]. This supports the idea that physical activity and engagement in sports can have a beneficial impact on ADHD symptoms.

The present study examined various aspects of social media use and their association with ADHD risk. The time spent on social media during different periods (study periods, weekends, and holidays) showed statistically significant differences in ADHD risk. Spending more hours on social media was associated with a higher risk of ADHD. Additionally, specific activities on social media, such as watching reels, were also associated with an increased risk of ADHD. These findings are consistent with previous research on the relationship between social media use and ADHD symptoms. A study conducted on a sample of adolescents [30] found that excessive use of social media was associated with higher levels of ADHD symptoms.

The multivariate analysis identified three significant independent factors affecting ADHD risk among Egyptian university students. Watching reels, spending more hours on social media during the study period, and practicing sports were found to be associated with ADHD risk. The finding that watching reels on social media was associated with increased ADHD risk aligns with a cross-sectional study conducted among Lebanese adults to measure the association between problematic social media use and Attention-Deficit/Hyperactivity [12].

### Limitations of the study

A potential limitation of this study is the lack of randomization in the sample selection, which may introduce selection bias and affect the generalizability of the findings. The authors added a design effect of 2.5 to the sample size and power analysis to deal with this defect and increase the sample size. Another limitation is the cross-sectional design of the study, which prevents causal inference and temporal analysis of the relationships among the variables. Therefore, further longitudinal studies are needed to confirm and extend the results of this study.

## Conclusion

Based on our analysis, several factors have been identified as potentially influential in the development of ADHD. Specifically, our findings indicate that engaging in activities such as watching reels, status updates, stories, and shorts on social media, as well as spending an increased number of hours on social media during the study period, are independent risk factors associated with ADHD. Conversely, practicing sports has emerged as an independent protective factor.

## Recommendations

It is important to note that further research is required to understand the complexity and interplay of these factors fully. In light of the present study findings, we recommend that parents remain vigilant in observing any potential symptoms of ADHD in their children encouraging children to participate in regular sports activities, and minimizing their exposure to short video formats like reels. Moreover, we propose that social media platforms implement restrictions on the availability and accessibility of such short video content across platforms like Instagram, Facebook, WhatsApp, YouTube, and TikTok. For university students, we encourage engaging in more interactive activities such as conversations with friends and information-seeking, rather than passive consumption of these short videos. Additionally, promoting regular participation in sports activities is recommended.

It is important to acknowledge that our study has identified some significant associations; however, further investigations are warranted to gain a more comprehensive understanding of the underlying mechanisms and potential causal relationships. Continued research in this area will contribute to a better understanding of the multifaceted nature of ADHD and inform effective prevention and intervention strategies.

## Data Availability

Data are available upon reasonable request from the corresponding author.
